# A deep look into the rib cage compression technique in mechanically ventilated patients: a narrative review

**DOI:** 10.5935/0103-507X.20220012-en

**Published:** 2022

**Authors:** Yorschua Jalil, L. Felipe Damiani, Roque Basoalto, María Consuelo Bachmman, Alejandro Bruhn

**Affiliations:** 1 Departamento de Medicina Intensiva, Facultad de Medicina, Pontificia Universidad Católica de Chile - Santiago, Chile.

**Keywords:** Physical therapy modalities, Respiration, artificial, Critical care, Respiratory therapy, Mucociliary clearance, Rib cage, Pressure

## Abstract

Defective management of secretions is one of the most frequent complications in invasive mechanically ventilated patients. Clearance of secretions through chest physiotherapy is a critical aspect of the treatment of these patients. Manual rib cage compression is one of the most practiced chest physiotherapy techniques in ventilated patients; however, its impact on clinical outcomes remains controversial due to methodological issues and poor understanding of its action. In this review, we present a detailed analysis of the physical principles involved in rib cage compression technique performance, as well as the physiological effects observed in experimental and clinical studies, which show that the use of brief and vigorous rib cage compression, based on increased expiratory flows (expiratory-inspiratory airflow difference of > 33L/minute), can improve mucus movement toward the glottis. On the other hand, the use of soft and gradual rib cage compression throughout the whole expiratory phase does not impact the expiratory flows, resulting in ineffective or undesired effects in some cases. More physiological studies are needed to understand the principles of the rib cage compression technique in ventilated humans. However, according to the evidence, rib cage compression has more potential benefits than risks, so its implementation should be promoted.

## INTRODUCTION

Invasive mechanically ventilated patients are at risk of several respiratory complications, with secretion retention being one of the most frequent.^(1-3)^ Clinically, secretion accumulation may cause bronchial obstruction and worsening of gas exchange and, in some critical cases, can affect ventilatory support performance,^(4,5)^ leading to a longer duration of mechanical ventilation (MV) and higher mortality.^(6-8)^

The endotracheal tube (ETT) across the airway may severely affect mucociliary transport by increasing the volume and viscosity of secretions, as well as predisposing the patient to respiratory infections.^(1,3,9)^ In addition, inadequate thermohumidification and the use of certain ventilatory modes and asymmetric air flow patterns may contribute to secretion accumulation.^(2,10,11)^ Moreover, the immobility of these patients, the use of sedative drugs and general muscle weakness, particularly the respiratory muscles, may impair the cough mechanism.^(12-14)^

Management of secretions is a critical aspect of the treatment of mechanically ventilated patients. Routine standard techniques to manage and counteract this problem include adequate airway humidification, endotracheal suctioning (ETS) and early physical mobilization. However, when these methods fail, because of the profuse amount of mucus or peripheral allocation, chest physiotherapy (CPT) can be provided through mechanical devices and/or manual techniques.^(8,15-17)^ On the other hand, conventional CPT is widely used because it does not require any device, does not need to disconnect the patient from the ventilator and is cheaper.^(8,18)^ Conventional CPT includes manual percussion, postural drainage and rib cage compression (RCC), with the latter being one of the most practiced CPT techniques in ventilated patients.^(19)^ However, its impact on clinical outcomes remains controversial due to methodological issues and poor understanding of its action.^(17,19)^ In this review, we aimed to comprehensively describe the physical principles of the RCC technique as well as its physiological effects in experimental and clinical studies.

### Rib cage compression technique

According to the literature, the RCC technique is also referred to as manual chest compression or “squeezing”,^(20,21)^ and its definition varies. In general, terms, the technique consists of a compression of the chest wall at the beginning of the expiratory phase and is aimed at simulating the final phase of coughing: the expulsive maneuver.^(20)^

The RCC pursues to promote greater air compression during expiration, increasing the expiratory flow and the displacement of the secretions toward the trachea, where they can be removed by coughing or tracheal suction.^(20,22-24)^ Classically, manual force is applied exclusively to the chest, placing the hands bilaterally on the lower third of the thorax.^(20,21,25)^ For a brief period of time (i.e., 1 or 2 seconds), the operator uses both hands to squeeze the ribcage during expiration, trying to include the most affected lung region.^(23)^

Rib cage compression is provided by registered nurses and physiotherapists. Some studies describe the application of a “gradual squeeze”, while others describe a “vigorous or hard compression” to the rib cage, showing different clinical outcomes.^(21,23,25,26)^ These technical features may influence RCC performance and its clinical impact.

First insights into manual chest compression functioning date from the 1950s, when Opie et al. hypothesized that local compression of the chest generates a “tooth-paste” effect by squeezing out the retained material through the bronchus.^(27)^ The mechanisms involved in this phenomenon captured the attention of other scientists, leading to new insights into mucous layer functioning and therapeutic strategies to enhance it.

### Physical principles of airway clearance

Three main factors seem to be critical for secretion transportation; cilia movement, gravity and interaction with the airflow.^(28)^ These last two factors are especially important in the first generations of the bronchial tree and trachea.^(28)^ The interaction between secretion movement and airflow is fundamentally explained by a two-phase gas-liquid (TPGL) flow model.^(29,30)^ The mucus movement (liquid phase) originating from the airflow stress (gas phase) over its surface is comparable to that achieved by gravity and cilia movement, suggesting that asymmetrical airflow profiles are responsible for outward mucus movement.^(28,31)^

#### *In vitro* studies

**Figure 1:**
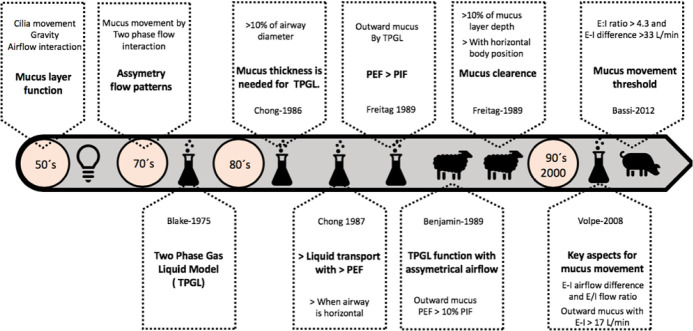
Timeline for physical principles of airway clearance. TPGL - two-phase gas-liquid; PEF - peak expiratory flows; PIF - peak inspiratory flow; E/I - expiration and inspiration flow.

Mucous layer transport in the respiratory airways by the TPGL mechanism has been studied under different conditions (Figure 1). Kim et al., using a vertical tube model, found that a critical thickness is needed to achieve mucus transport by airflow interaction. In this experiment, the authors found that a thin mucous layer less than 10% of the diameter of the tube could not be effectively transported by the TPGL mechanism, which seems to have poor relevance to normal lung situations *in vivo* where the mucus layer is usually extremely thin. However, the rate of mucus production is usually higher in disease and often exceeds several hundred milliliters, resulting frequently in substantial mucus accumulation and the consequent occupation of approximately 5 to 20% of the airway diameter. In addition, the vertical tube represents the worst condition for mucous layer displacement, being far from the clinical setting where patients are in a semirecumbent position.^(32)^ The airways are usually inclined, and the directions of cephalad flow in many airway branches are even downward, being a more favorable condition for mucus transport that may be achieved *in vivo*. However, it must be recognized that this experimental model does not closely simulate the complex nature of airway flow *in vivo*, where many factors need to be cautiously extrapolated.^(32)^

Kim et al., in another report, studied the impact of asymmetric flow patterns on mucus transport when the tube was placed either vertically or horizontally. They found that the speed of liquid layer transport increased with higher peak expiratory flows (PEFs), but inspiratory flow rate, respiratory rate and tidal volume had no impact. In the vertical tube, the upward transport of mucus could not be achieved at a PEF lower than 30L/minute. Under similar conditions, in the horizontal tube, the mucus transport speed was 5% to 60% faster. However, at higher values of PEF, the mucus transport speed in the vertical position was comparable to that attained in the horizontal position.^(29)^ These results suggest that a flow pattern with higher expiratory than inspiratory peak flows must be obtained to observe an outward mucus displacement by gas-liquid interaction, a strictly physical phenomenon that will occur regardless of *in vivo* or *in vitro* situations if the basic requirements are met, namely, a sufficient amount of mucus and asymmetric airflow pattern favoring an expiratory airflow.^(33)^

Volpe et al., with a test-lung system, revealed that the proportion between expiration and inspiration (E/I) flow ratio and the airflow difference between expiration and inspiration (E-I) were important to mucus movement. However, the E-I difference showed a stronger correlation with the displacement of mucus of different viscosities. An E-I flow difference of > 17L/minute seems to be a threshold to change the direction of mucus movement toward the mouth.^(34)^

#### *In vivo* studies

Benjamin et al. developed an animal model with sheep connected to MV using the following three settings: inspiratory time/total time proportion (Ti/ Tt) of approximately 0.27, 0.65 and 0.75, and they found outward mucus movement with the two latter configurations. According to this, mucus will move when the expiratory time is shorter than the inspiratory time. This is based on expiratory flow velocity, which needs to be higher in order to expulse the same amount of air volume that entered during inspiration. They found that mucus displacement remained unaffected when the peak inspiratory flow rate differed by less than 10% from the PEF. The asymmetric airflow found in breathing causes unequal flow velocities and unequal shear forces in opposing directions; therefore, the liquid layer will move according to the difference in airflow velocity between the two phases, not only based on PEF.^(35)^

Freitag et al. ventilated animals using the following two MV settings: expiratory bias (PEF 3.8L/s and peak inspiratory flow 1.3L/s) and inspiratory bias (PEF 1.3L/s and peak inspiratory flow 3.8L/s). Different body positions were assessed (horizontal, prone, lateral decubitus and head-down tilt). In the horizontal position, mucus clearance with expiratory bias increased significantly in comparison to inspiratory bias, in which no clearance occurred even during head-down tilt. This observation suggests that expiratory bias airflow might be the dominant factor in clearing mucus, which can be augmented by postural drainage.^(36)^ Another interesting finding was that because of the uneven surface of the trachea, the minimum mucus layer depth required for TPGL transport *in vivo* is much greater than that predicted by the tube models, suggesting that similar conditions are needed in intubated humans.^(36)^

Li Bassi et al. conducted a prospective randomized study to evaluate the effects of duty cycles and positive end-expiratory pressure (PEEP) on mucus clearance in ventilated pigs. Six levels of duty cycle were administered (inspiration time/total time; 0.26, 0.33, 0.41, 0.50, 0.60, and 0.75) with either 0 or 5cmH_2_O of PEEP. Beds were oriented in the semirecumbent position, emulating usual clinical practice. No effect from PEEP was found, but as the duty cycle was prolonged (shorter total time; same volume of inspired gas has shorter time to be exhaled), a trend in increasing outward mucus transport speed was found. In this context, a duty cycle of > 0.41 is proposed as a threshold since an increased mean E-I flow bias difference was associated with an increased outward mucus velocity.^(37)^

### Rib cage compression impact

#### Experimental studies

Considering the physical mechanisms of airway clearance, it seems that the effect of RCC may depend on the magnitude of E-I flow difference, airway position, mucus viscosity and its location in the bronchial tree.

Unoki et al. studied the effects of RCC and/or prone position on gas exchange in mechanically ventilated rabbits with atelectatic lung injury.^(21)^ Animals were allocated to one of the following four groups: supine without RCC, supine with RCC, prone without RCC, and prone with RCC. The RCC group did not experience sustained improvement in oxygenation, dynamic compliance or mucus output.^(21)^ The relatively high viscosity of mucus and inadequate RCC technique could explain the poor effect on mucus transport. The RCC was described as gradually squeezing until the end of expiration, which was applied by a single operator who used pressure applied to the rib cage as an indicator of technique performance; however, no attention was given to airflow magnitude, its main determinant. The collapsed lung units, as part of the atelectasis model, might not be recruited, affecting pulmonary volume and with this impairing the potential expiratory flow, which is necessary to remove the mucus plugs in the airways and increase transpulmonary pressure, causing a vicious cycle of damaging the lungs and effecting their mechanics.^(21)^ Unoki et al., in another study with similar methods, evaluated the effects of “gradual” RCC with and without ETS on gas exchange, dynamic compliance, and mucus clearance. The authors observed that in the RCC groups, gas exchange and compliance were significantly worse than those without RCC. In addition, no differences in the aspirated mucus weight were found between groups, concluding that alveolar and airway collapse was probably exacerbated by RCC.^(38)^ Notwithstanding, it is necessary to consider that RCC was applied with zero PEEP to “avoid the effects of PEEP on expiratory flow during RCC”.

In contrast with previous evidence, Martí et al. tested a hard and brief RCC (with early expiratory phase) and a soft and gradual RCC (applied during late expiratory phase) over expiratory flow and mucus clearance in mechanically ventilated pigs.^(26)^ Mean expiratory flow increased significantly with hard RCC compared to soft RCC. During hard RCC, mucus moved toward the glottis, while the application of soft RCC or no intervention moved mucus toward the lungs. Additionally, they showed that soft RCC, which is performed from mid-low pulmonary volume (mid-expiratory phase), slightly worsened static lung elastance, the reciprocal of compliance, in part due to decreased PEEP levels resulting from prolonged compression looking for final zero flow and consequently prolonged expiratory time.^(26)^ These findings corroborate the predominant role of expiratory flow on mucus clearance, making evident that its measurement is a critical aspect to its performance. Ouchi et al. found that during hard RCC, the mean PEF increased compared with no treatment and that combined with endotracheal suctioning, mucus clearance increased in comparison with ETS alone. However, no improvement in gas exchange was found.^(39)^

It seems that the RCC technique is critical to determine the effects on secretion movement. Hard RCC shares similarities to the huffing technique or forced expiration technique, which was originally designed to rapidly increase the expiratory flow rate from mid-to-low lung volumes.^(26,40)^ On the other hand, soft and gradual RCC is comparable to the prolonged slow expiration technique, which is applied during the late phase of expiration up to the residual volume to improve the interaction of the expiratory airflow with the mucus layer, specifically within the distal narrower airways.^(26,41,42)^ However, in affected lungs, excessive compression over the whole expiratory phase can impair the residual lung volume, explaining some of the negative findings regarding mucus removal and compliance when soft RCC is applied.

#### Clinical studies

The impact of RCC on different clinical outcomes has been studied under several conditions. Factors including the type of RCC technique, expiratory airflow and ventilator setting seem to have a key role. Table 1 summarizes the clinical evidence regarding these topics, focused on clinical trials, since this approach provides a higher level of certainty from an interventional point of view.

**Table 1:** Rib cage compression clinical evidence

Author	Study design	Population	RCC feature	Results regarding RCC	Limitations
Avena et al.^(20)^	Prospective randomized study	16 mechanically ventilated patients	Hard and brief RCC	No differences in peak inspiratory pressure, plateau pressure, dynamic or static compliance Decrease in pulmonary and airway resistances, as well as an increase in oxygen saturation	No airflow measurement, volume of secretions or relation between ventilatory setting and RCC was reported
Unoki et al.^(21)^	Prospective randomized study	40 mechanically ventilated rabbits with induced atelectasis	Soft and gradual RCC	No improvement on oxygenation, dynamic compliance, or mucus output	No airflow measurement
Kohan et al.^(23)^	Randomized crossover trial	70 mechanically ventilated patients	Soft and gradual RCC	Gas exchange was significantly different from the baseline RCC determined a significant improvement in oxygenation	The patients’ respiratory pathophysiologies were not uniform
Bousarri et al.^(24)^	Randomized crossover trial	50 mechanically ventilated patients	Soft and gradual RCC	An increase in vital signs within a normal range	No limitation or any complication was reported
Unoki et al.^(25)^	Randomized crossover trial	31 mechanically ventilated patients	Soft and gradual RCC	No significant differences in gas exchange, dynamic compliance, and secretion removal	The patients’ respiratory pathophysiology which led to mechanical ventilation was not uniform
Martí et al.^(26)^	Prospective randomized study	9 mechanically ventilated pigs.	Hard and brief RCC Soft and gradual RCC	With hard RCC greater mean expiratory flow and mucus moved toward the glottis With soft RCC mucus moved toward the lungs	The interventions were conducted by a single respiratory physiotherapist
Unoki et al.^(38)^	Prospective randomized study	24 mechanically ventilated rabbits with induced atelectasis	Soft and gradual RCC	Oxygenation, ventilation, and compliance were significantly worse No significant differences in the weight of aspirated artificial mucus	Along with RCC, a PEEP zero intervention was added Anatomic and physiologic differences between rabbits and humans
Ouchi et al.^(39)^	Prospective randomized study	15 mechanically ventilated pigs with induced atelectasis	Hard and brief RCC	Greater peak expiratory flow and mucus removal Not improve in gas exchange or radiologic findings	The diagnosis of atelectasis may have lacked optimal sensitivity
Sixel et al.^(43)^	Randomized crossover trial	20 mechanically ventilated patients with pulmonary infection	Soft and gradual RCC (none explicated)	34.4% more secretions cleared No differences in static or effective compliance, total or initial resistance PEF and expiratory flow at 30% of expiratory tidal volume significatively increased	Effect size was small for secretion removal and compliance, and negligible for resistance Six subjects presented expiratory flow limitation
Gonçalves et al.^(44)^	Randomized crossover trial	30 mechanically ventilated patients	Hard and brief RCC (none explicated)	More secretions were removed. No difference for gas exchange or pulmonary mechanics	No detailed intervention neither the number of subjects in each group of analysis were provided

RCC - rib cage compression; PEEP - positive end-expiratory pressure; PEF - peak expiratory flow.

Unoky et al. studied the effects of RCC on airway secretion removal, oxygenation, and ventilation in thirtyone mechanically ventilated patients who were randomized to ETS with or without RCC. No airflow measurement was made during RCC, which was performed by trained nurses, who used both hands to gradually squeeze the rib cage during expiration. There were no significant differences in gas exchange, dynamic compliance or secretion removal.^(25)^ In contrast, in a similar context, but using vigorous chest compression, Avena et al. showed that post RCC and ETS, a significant decrease was observed in pulmonary and airway resistances, as well as an increase in oxygen saturation (SpO_2_) compared with ETS only.

However, no differences were observed in peak inspiratory pressure (PIP), plateau pressure (Pplat), dynamic or static compliance (Cst).^(20)^ These encouraging clinical results advocate for a safe and efficient technique for this population. Unfortunately, airflow measurement, volume of secretions and the relationship between ventilatory setting and RCC were not reported, despite the significant evidence about the relevance of these factors.^(2,11,34)^

A randomized crossover study performed by Sixel et al. evaluated the mechanical and sputum removal effects of RCC in comparison to control intervention in twenty ventilated patients with pulmonary infection. They found that RCC cleared 34.4% more secretions than the control and that there were no differences after intervention in terms of Cst, effective compliance (Ceff), total resistance (Rtot), and initial resistance (Rinit). However, the effect size was small for secretion removal, Cst, and Ceff and negligible for Rtot and Rinit, limiting the clinical interpretation of these findings.^(43)^ During RCC, PEF and expiratory flow at 30% of expiratory tidal volume significantly increased (16.2L/minute and 25.3L/ minute, respectively) compared to the control, which is an important feature but not as critical as the E-I airflow difference. Considering that inspiratory flow was set at 60L/minute and baseline PEF was 43.6 ± 17.5, which increased with RCC to 59.6 ± 18.3, an E-I magnitude of < 17L/minute was achieved in most patients, well below the threshold previously mentioned to achieve effective mucus movement. Unfortunately, no technical details of the RCC applied were provided, during which six subjects presented expiratory flow limitation (EFL).^(43)^ As Martí commented, given these side effects, it is possible that compressions were applied through the whole expiratory phase, favoring this phenomenon.^(45)^ Martí reported a transitory loss of PEEP of approximately 3cmH_2_O associated with prolonged compression, reinforcing the notion that critical factors, including technique features, should be considered.^(45)^

A couple of reports assessed the effects of RCC prior to ETS in terms of blood gases and vital signs. Kohan et al. showed that gas exchange at 25 minutes after RCC or ETS alone was significantly different from baseline. Interestingly, when comparing RCC with only ETS, the first one determined a significant improvement in oxygenation.^(23)^ Bousarri et al., in a similar study, showed that vital signs during ETS with RCC remained in normal ranges.^(24)^ In both reports, gradual RCC was applied, and no events or signs of flow limitation were reported.

Gonçalves et al. in a randomized crossover clinical trial with thirty subjects in controlled MV who were randomized to control (placebo and ETS) or RCC in patients classified into a no secretion group (NSG; £ 2g) and secretion group (SG;^^3^^ 2g).^(44)^ The authors observed that with RCC, more secretions were removed. No difference was found for gas exchange or lung mechanics between groups, except for slight improvement in static compliance in the SG who received RCC.^(44)^ It seems possible, according to the physical principles described above, that the proportion of airways with secretions and the viscosity of secretions might affect the optimal response to RCC in some patients, which is of great clinical importance to decide which patients can be “RCC responders”.

#### Potentiated rib cage compression

An alternative form of the RCC technique may also be employed in particular scenarios. Some authors describe the use of abdominal compression (in a cephalic way) simultaneously with chest compression to mimic the normal movement of the diaphragm during a cough, more efficiently managing intraabdominal pressure.^(20,46,47)^ This has been reported mostly for patients with neuromuscular diseases or with a condition leading to abdominal muscle weakness (i.e., sedated/paralyzed patients receiving MV at the ICU).^(46,47)^ However, in patients requiring controlled MV, the application of abdominalthoracic compression has not shown differences in terms of PEF augmentation compared with RCC alone.^(48)^

Another maneuver closely related to RCC is positive end expiratory pressure - zero end-expiratory pressure (PEEP-ZEEP). Theoretically, when PEEP rises, the gas is redistributed through collateral ventilation, therefore, reaching adjacent alveoli previously collapsed by mucus. This redistribution favors reopening of small airways by removing the mucus adhering to their walls. Later, in a subsequent phase of the technique when PEEP is reduced to 0cmH_2_O, the expiratory flow pattern is changed aiding transport of secretions from peripheral to central airways.^(49)^ Santos et al., in a crossover study comparing the effects of RCC versus the PEEP-ZEEP maneuver in ventilated patients, found that both interventions had positive clinical effects on tidal volume and static and dynamic compliance without differences between groups except for oxygenation (SpO2), which was favorable to the RCC group.^(49)^ Lobo et al. compared PEEP-ZEEP plus vibrocompression (not RCC alone) against bag squeezing (manual hyperinflation), showing that both techniques are similar regarding bronchial secretion removal and hemodynamic changes during their use.^(50)^ In a similar study, Oliveira et al. reported that the PEEP-ZEEP maneuver without RCC was enough to exceed the E-I airflow difference of 33L/ minute (secretion move threshold). However, using RCC with PEEP-ZEEP, this difference increased by 6.7 ± 3.4L/ minute, which could improve their potential for secretion removal.(51)

To date, these mixed techniques are feasible and could potentiate the effect of RCC alone; however, the level of evidence remains low.

#### Summary

**Figure 2:**
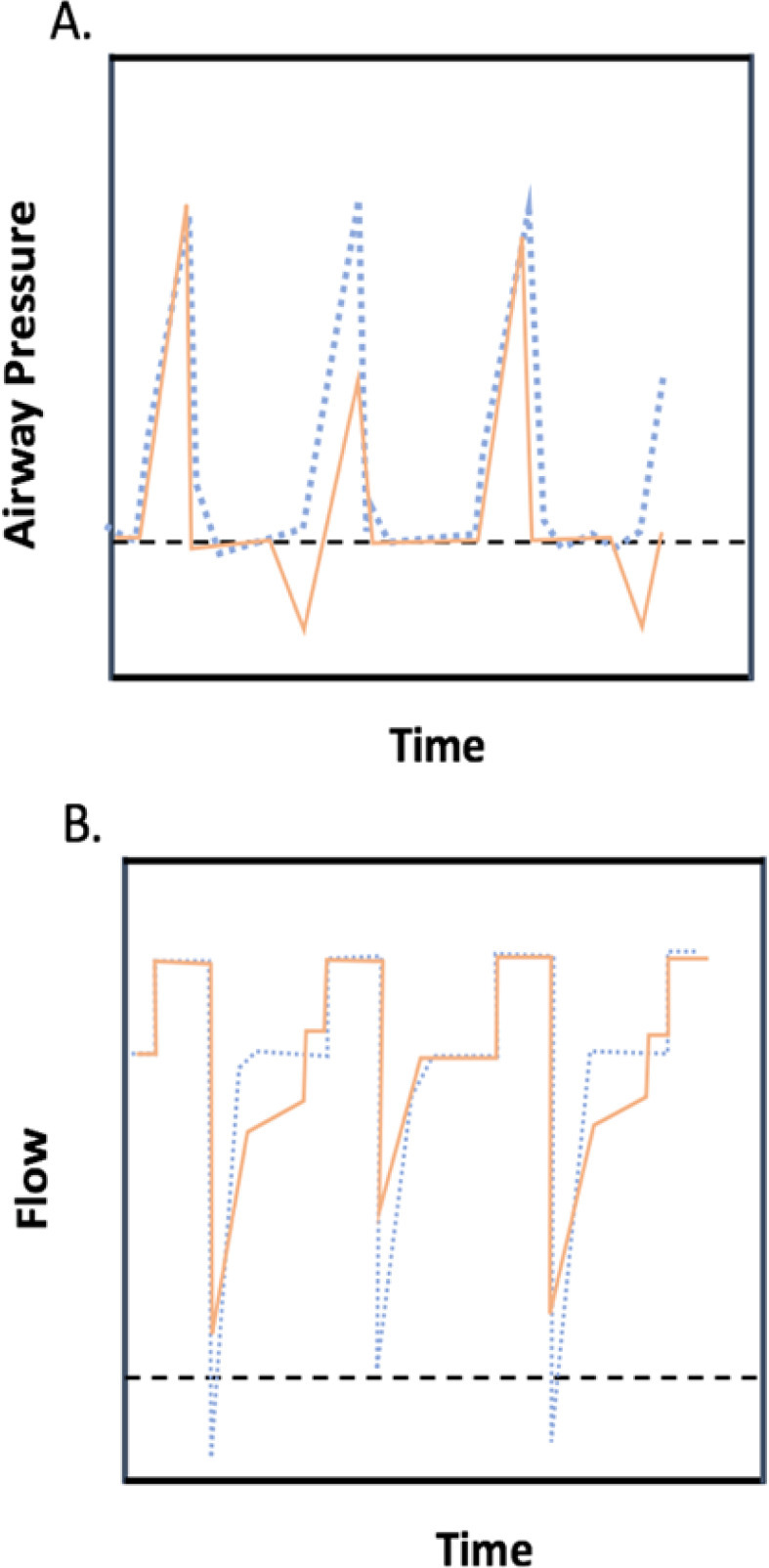
Representation of hard versus soft rib cage compression. (A) Black dotted line indicates expiration and inspiration flow level. (B) Airflow/time curve. Black dotted line indicates airflow without treatment. Blue dotted line indicates hard rib cage compression and red indicates soft rib cage compression.

Borges et al. published in 2017 a systematic review with meta-analysis about expiratory RCC in mechanically ventilated adults concluding that there is lack of evidence to support the use of expiratory RCC in routine care.^(52)^ They included three studies in their final analysis; the studies of Unoki et al.,^(25)^ Bousarri et al.^(24)^ and Santos et al.^(49)^ All of them discussed in the present review had different outcome measures and RCC technique features. We know that the evidence is heterogeneous, probably because the nature of this intervention is complex, and the fact that it is commonly inserted in a multimodal therapy provided by CPT where mechanisms involved are not yet completely elucidated. Spapen et al. indicated, in their narrative review about CPT in mechanically ventilated patients, that “RCC is best supported by experimental and preliminary clinical experience”.^(19)^ The detailed analysis presented in this review, which goes from physical principles of action, *in vitro* testing and animal models, through human implementation, supports the use of a brief and vigorous RCC as an airway clearance technique based on mucus displacement by increased expiratory flows (E-I airflow difference). On the other hand, soft and gradual RCC through the whole expiratory cycle does not improve PEF or mucus output and could be related to a PEEP decrease and airflow limitation as an undesired effect (Figure 2). However, clinical trials directly comparing these two interventions are needed to support this approach.

## FINAL COMMENTS

The thresholds to achieve the displacement of secretions in the correct direction have been clearly established and therefore should be actively sought through mechanic ventilator monitoring using time cycles, peak flow values and graphic trends to guide proper RCC implementation. However, factors such as the viscosity of the secretions and the occupation ratio of the tracheal lumen unfortunately still remain elusive to assess in clinical practice. Rib cage compression can be enhanced by other maneuvers, such as PEEP-ZEEP and abdominal compression; nevertheless, more studies should be conducted to justify its inclusion routinely in the respiratory care of patients with ventilatory support. There is no doubt that more physiological studies are needed to better understand the mechanisms involved in the RCC technique in ventilated humans as well as clinically relevant evidence regarding its impact on MV use and intensive care unit stay. However, according to the evidence presented, RCC has more potential benefits than deleterious effects, and its implementation should not be limited. The opposite is true, it should be recommended, considering that it is one of the few strategies to avoid the retention of secretions in the critical care setting.
