# Erratum

**DOI:** 10.5935/0103-507X.20220012-er-en

**Published:** 2022

In the article **A deep look into the rib cage compression technique in mechanically ventilated patients: a narrative review**, with DOI number: 10.5935/0103-507X.20220012-en, published in the journal Revista Brasileira de Terapia Intensiva, 34(1):176-84, on page 180 - Table 1 - Ribe cage compression clinical evidence and in the second pafagraph:

**Where it read**:

Sixel et al.^(43)^

**Read**:

Guimarães et al.^(43)^

In the same article, page 183 - References:

**Where it read**:

43. Sixel BS, Lemes DA, Pereira KA, Guimarães FS. Compressão manual torácica em pacientes com insuficiência respiratória aguda. Fisioter Bras. 2007;8(2):103-6.

**Read**:

43. Guimarães FS, Lopes AJ, Constantino SS, Lima JC, Canuto P, de Menezes SL. Expiratory rib cage compression in mechanically ventilated subjects: a randomized crossover trial. Respir Care. 2014;59(5):678-85.

